# Dynamic peripheral nerve stimulation can produce cortical activation similar to punctate mechanical stimuli

**DOI:** 10.3389/fnhum.2023.1083307

**Published:** 2023-03-24

**Authors:** Justin Tanner, Edward Keefer, Jonathan Cheng, Stephen Helms Tillery

**Affiliations:** ^1^School of Biological and Health Systems Engineering, Arizona State University, Tempe, AZ, United States; ^2^Nerves Inc., Dallas, TX, United States; ^3^University of Texas Southwestern Medical Center, Dallas, TX, United States

**Keywords:** peripheral nerve stimulation, gamma, somatosensory feedback, biomimetic stimulation, perception

## Abstract

During contact, phasic and tonic responses provide feedback that is used for task performance and perceptual processes. These disparate temporal dynamics are carried in peripheral nerves, and produce overlapping signals in cortex. Using longitudinal intrafascicular electrodes inserted into the median nerve of a nonhuman primate, we delivered composite stimulation consisting of onset and release bursts to capture rapidly adapting responses and sustained stochastic stimulation to capture the ongoing response of slowly adapting receptors. To measure the stimulation’s effectiveness in producing natural responses, we monitored the local field potential in somatosensory cortex. We compared the cortical responses to peripheral nerve stimulation and vibrotactile/punctate stimulation of the fingertip, with particular focus on gamma band (30–65 Hz) responses. We found that vibrotactile stimulation produces consistently phase locked gamma throughout the duration of the stimulation. By contrast, punctate stimulation responses were phase locked at the onset and release of stimulation, but activity maintained through the stimulation was not phase locked. Using these responses as guideposts for assessing the response to the peripheral nerve stimulation, we found that constant frequency stimulation produced continual phase locking, whereas composite stimulation produced gamma enhancement throughout the stimulus, phase locked only at the onset and release of the stimulus. We describe this response as an “Appropriate Response in the gamma band” (ARγ), a trend seen in other sensory systems. Our demonstration is the first shown for intracortical somatosensory local field potentials. We argue that this stimulation paradigm produces a more biomimetic response in somatosensory cortex and is more likely to produce naturalistic sensations for readily usable neuroprosthetic feedback.

## 1. Introduction

Tactile information is paramount in learning and executing fine motor tasks, integrating with and even prioritized over visual stimuli (Marsden et al., 1984; Ernst and Banks, 2002; Forster et al., 2002; Diederich and Colonius, 2004). As such, quality somatosensory feedback is a persistently desired and a needed factor of neuroprosthetic adoption and practical use. Provision of tactile feedback is increasingly prioritized with improvements in myoelectric devices (Biddiss and Chau, 2007; Peerdeman et al., 2011).

Efforts to deliver reliable feedback have yielded suboptimal outcomes. Vibrotactile stimulation can benefit proprioceptive estimation, but subjects are not inherently good at detecting differences in vibration intensity or frequency (Mann, 1973). Punctate force feedback on a residual limb provides improvement in task performance, but not to normal levels (Meek et al., 1989). Even peripheral nerve stimulation with localized and graded sensations produces results that leave much room for improvement as the resulting percepts are typically incongruous with natural sensations. Standard stimulation strategies consist of repeating charge-balanced, square pulses at defined magnitude, pulse width, and frequency. Sensations can be manipulated by varying these stimulus parameters. At lower stimulation frequencies, sensations are reminiscent of tingling/paresthesia. Increasing frequency progresses the subject through tapping, pulsing, vibration, possible pressure, and eventually pain responses (Clippinger et al., 1974; Walker et al., 1977; Gasson et al., 2005). Modulating parameters provides mediocre feedback, still with vibration, tingling, or pulsing percepts (Dhillon and Horch, 2005), although these sensations have been reported as “pleasant and emotionally satisfying” to the subject (Clark et al., 2014). In addition to modulating parameters, specific electrode design can improve discrimination as well. Subjects with implanted fascicle-specific targeting of longitudinal intra-fascicular electrodes (FAST-LIFEs) feel a wide range of sensations with variable percept size. This electrode design provides high stability, increased recruitment specificity, and the opportunity for increased quality of sensations (Overstreet et al., 2019).

Feedback stimulation which achieves sensations felt in natural tasks would help overcome the existing hindrances to prosthetic acceptance. The properties necessary for such stimulation remain unclear but would likely require incorporating the complex dynamics observed in natural neural responses. Physiologically, subject reactions to and perceptions of weight, texture, and movement are based on the shear force distribution on the finger pads and how that varies with respect to the task context (Westling and Johansson, 1984; Häger-Ross et al., 1996; Wang and Hayward, 2007; Seizova-Cajic et al., 2014). Subjects use these variables to intentionally adjust and plan anticipatory force loadings for new grips (Forssberg et al., 1992; Chang et al., 2008; Fu et al., 2010). Any initial errors are corrected by immediate tonic and phasic mechanoreceptor activity, (Johansson and Westling, 1987; Westling, 1987) similar to and preceding the bimodal activity common in the peripheral nerves and the somatosensory cortex (Salimi et al., 1999a,b,c).

Using time-variant stimulation properties, graded, precise percepts that provide a natural sensation have been achieved. In bidirectional prosthesis usage, varying current as a function of sensor activation or varying stimulation pulse width provides the ability to successfully grade prosthetic use (Raspopovic et al., 2014; Tan et al., 2014). In one such study, subjects specifically reported the sensations “as natural as could be (Tan et al., 2014).” Both patterns are effectively delivering temporal variance of stimulation charge in two modes: onset-release phasic patterns associated with the rate of force from a sensor or sustained tonic stimulation with dynamic properties. Time variant patterns offer a promising path toward natural sensation, but with vague suggestions toward improvement and understanding.

Measures for perception are needed in order to quantify differences and benefits in feedback sensations, particularly during the development of neurotechnologies when many of our conclusions are drawn from non-human animals. One possible measure lies in the change in cortical frequency bands’ power and phase locking state during sensory stimulation. Cortical activity can be segregated into three bands of frequencies: alpha at 8–12 Hz, beta at 14–28 Hz, and gamma at 30 to 100 Hz. After a stimulus, the first band to respond is gamma, showing activity within 20 ms, followed by activity in the beta and then alpha bands (Fries, 2009). When subjects are paying attention to a stimulus, gamma augmentation can scale with either attention to a stimulus or the perceived stimulus intensity at a latency of ∼250 ms. Perceptual gamma augmentation at ∼250 ms is congruous with the established P300 response observed in various abstract (Wood et al., 1980; Duncan-Johnson and Donchin, 1982; Gray et al., 2004; Yang and Zhang, 2009) and tactile recognition (Yamaguchi and Knight, 1991) tasks associated with cortical recognition of a stimulus and the “top down” processing of sensory inputs. This response is present in cortical structures related to auditory (Jokeit and Makeig, 1994; Bertrand et al., 1998; Crone et al., 2001; Gurtubay et al., 2004; Roth et al., 2013), visual (Goertz et al., 1994; Tallon-Baudry et al., 1996, 1997; Slobounov et al., 2000; Goffaux et al., 2004), electrotactile (Fukuda et al., 2008; Rossiter et al., 2013; Wittenberg et al., 2018), laser induced pain (Gross et al., 2007; Zhang et al., 2012; Bassez et al., 2020), peripheral nerve stimulation (Babiloni et al., 2001), or tactile stimuli (Bauer et al., 2006; van Ede et al., 2014). Tactile sensitivity can even be heightened by artificially raising baseline gamma (Tan et al., 2019). Attention-modulated augmentation of gamma to tactile stimuli has only been shown in magnetoencephalography (MEG) experiments, but MEG and EEG experiments in other sensory systems have both produced similar results. As well, intensity encoded nociceptive gamma band oscillations on the scalp have been related to spiking interneurons of S1 (Yue et al., 2020).

Activity in frequency bands can also be characterized in terms of phase locking. Phase locked gamma signals are consistent in latency, locked to stimuli across trials with little variance. Calculating the average Phase Lock Value (PLV) between trials (Lachaux et al., 1999; Rodriguez et al., 1999) for the gamma band splits it into separate phase locked components. Significant time-series PLV indicates a response locked to stimuli, while non-significant PLV implies the response possesses jittered latency between trials (Tallon-Baudry and Bertrand, 1999; Gross et al., 2007; Roach and Mathalon, 2008; Zhang et al., 2012). Inter-trial phase locking of the gamma band provides interesting insights with respect to perception. Depending on a subject’s attention to a sensory stimulus, distinct augmentations occur in both the non-phase locked and phase locked gamma between 30 and 65 Hz. In tasks where subjects receive a stimulus that they are not trained to recognize or respond to, only low latency phase locked gamma is present; however, when the subject is trained to respond to the same input, non-phase locked gamma is augmented at approximately 250 ms. In similar tasks with a distraction component where the subject must make a perceived choice based on the inputs, non-phase locked gamma is present for all stimuli but enhanced for the target stimulus. This occurs in studies utilizing auditory, visual, or tactile stimuli (Tallon-Baudry et al., 1996, 1997; Yordanova et al., 1997; Goffaux et al., 2004; Gurtubay et al., 2004; Bauer et al., 2006). Identifying how these cortical responses are dependent upon subject attention and stimulus intensity helps lay the groundwork to measure differences in perception. These observations suggest how the cortex should respond to useful and normal sensations: onset and release evoked potentials, onset phase locked gamma, and delayed non-phase locked gamma that scales to perceived intensity.

It is important to define other non-ordinary responses within the same framework. Painful sensations induced *via* median nerve stimulation, painful sensations induced *via* electrotactile stimulation, and non-painful sensations induced *via* electrotactile stimulation produce delayed phase locked gamma at lag times in which non-painful stimulations produce non-phase locked gamma (Babiloni et al., 2001; Fukuda et al., 2008; Rossiter et al., 2013). These are the only modes of somatotopic stimulation in which delayed phase locking in gamma has been observed, and all relied on consistent frequency stimulation schemes. There is also evidence for consistent delayed evoked potentials due to vibration, although frequency responses were not reported (Johnson et al., 1980). The outlier to this is seen in painful laser induced stimuli, which provides similar augmentation of intensity scaled delayed gamma, but it is not phase locked (Gross et al., 2007; Zhang et al., 2012). It is likely that the mechanism of sensation for laser induced percepts is distinct from mechanical or electrical stimuli. The presence of phase locked gamma activity at ∼250 ms after most sensory stimuli provides a cortical response metric that can differentiate aspects of perception. There is no gamma phase locking around 250 ms after stimuli in normal sensory perception, but it is present in the response to pain sensations from peripheral nerve stimulation.

These patterns of gamma activity provide a rubric to infer perceptual differences from cortical responses to various stimuli. Seeing phase locked gamma activity at the onset of stimulation and determining the gamma band’s phase lock state near the P300 point allows us to create a reference from punctate and vibrotactile stimulations with known sensations. From that, we can compare peripheral nerve stimulation patterns to determine what aspects of the stimulation pattern provide cortical responses more like the response to tactile stimulation.

Defining these differences in cortical response between stimulation modes will allow for the evaluation of different peripheral nerve stimulation patterns. Mechanical punctate stimulation can be used to represent a practical pressure percept that one would normally feel in tactile tasks. Vibrotactile stimulation can provide cortical responses associated with impractical vibration percepts, similar to those commonly observed from standard stimulation patterns. It is our goal to explore the effect that non-standard stimulation patterns have on the cortical response. Time variant patterns have produced promising results, and we proposed using a stimulation pattern based on the simultaneous onset-release bursts and stochastic firing seen in peripheral and cortical responses to touch (Johansson and Westling, 1987; Salimi et al., 1999a,b,c). Although separated into static and dynamic responses, neither the peripheral nor the cortical responses are truly compartmentalized nor are they consistent in timing or magnitude: stochasticity is present in all cortical signals. Deterministic neuron models do demonstrate appropriate phase locking to input, but models that include noise are more accurate predictors of cell sensitivity and activity (Bulsara et al., 1991; Longtin, 1993). Integrating this noise into the tonic portion of a stimulus increases resemblance to physiological representations, but it is unclear what effect that will have on detectability (due to stochastic facilitation) or phase locking (due to observations in cases of pathologic neural noise). The addition of stochastic properties to a stimulus are well investigated in terms of stochastic facilitation, where a subthreshold noise signal can randomly push a primary signal over some detection criterion (Benzi et al., 1981). In the tactile system, stochastic noise as vibration can increase detection of small physical geometries, and the cortical response exhibits increased gamma synchronization. However, suprathreshold noise can mask these previously detectable percepts (Collins et al., 1997; Ward et al., 2006). In addition, subjects with pathological neural noise show reductions in phase locked gamma responses to auditory tasks, even during the onset of auditory stimuli (Winterer et al., 2000; Roach and Mathalon, 2008; Roach et al., 2019; Rürup et al., 2020). While reducing phase locking is important near the P300 window, the phase locking at onset is still a necessary component of the ideal response. A balance of stochasticity is important in two ways: first, to increase perceptual sensitivity to a primary stimulus without masking the stimulus completely; and second, to control the suppression and augmentation of onset and delayed phase locking.

In the pursuit of improving artificial sensations for neuroprosthetic somatosensory feedback, this study aims to explore the effect which non-standard peripheral nerve stimulation patterns have on the cortical response of somatosensory cortex. Specifically, we suggest that gamma band power and phase-locking state of neural responses are well-supported cortical metrics of sensory perception seen in auditory, visual, and somatosensory systems. Applying those metrics, the cortical response to punctate stimulation serves as the benchmark for measuring the response to peripheral nerve stimulation. An ideal stimulation should produce phase locked gamma activity at the onset of stimulation, and non-phase-locked gamma activity at ∼250 ms that scales with the stimulus intensity. Vibrotactile stimulation elicits phase locking through the P300 window, and thus fails to reach this benchmark. We show that standard stimulation trains of constant frequency demonstrate similar responses to the vibrotactile stimulation, and increasing amplitude scales the phase-locked gamma activity response. Stimulation patterns with increased interpulse timing stochasticity still support an enhanced gamma response, but with decreased phase locking. Onset-release bursts elicit distinct responses to each burst, with little to no delayed response. Thus, combining stochastic sustained stimulation and onset-release bursts elicits separate key features of the cortical response to punctate stimulation.

## 2. Materials and methods

### 2.1. Overview

A single *Macaca mulatta*, Non-human primate R (NHP-R), was involved in this experiment. Cortical arrays were implanted into somatosensory cortex of the right hemisphere and intrafascicular nerve arrays in the contralateral median nerve. Mechanical and peripheral stimulation was passive, without the need for NHP-R feedback. Arizona State University Institutional Animal Care and Use Committee approve experimental protocols. The Arizona State University Department of Animal Care and Technologies provide veterinary supervision and care for all surgeries.

### 2.2. Experimental paradigm

Punctate, vibrotactile, and all peripheral nerve stimulation were delivered in similar experimental paradigms. A single location, either a fingertip or a stimulation electrode, was chosen. For peripheral nerve stimulation, the appropriate current amplitude was calculated from the determined activity thresholds, discussed later within the Section “3.5. Stimulation.” Each level of associated stimulation was repeated five times and the overall set delivered in randomized order with a variable intertrial interval between 5–8 s. For peripheral nerve stimulation, this was repeated ten times resulting in 50 trials (*n* = 50). For mechanical stimulation, there were nine punctate sessions (*n* = 45), and five vibrotactile sessions (*n* = 25). Recording of all stimulation patterns involved a 1 s pre-stimulus window, a half second of stimulation, and a half second post-stimulus window. For the task, NHP-R was restrained, and stimulation passively applied. Each trial resulted in a juice reward, and any trials where NHP-R was agitated or caused movement artifact were immediately discarded.

### 2.3. Electrode implantation

Somatosensory areas 1 and 3b in the right hemisphere of NHP-R were targeted for electrode implantation using stereotactic atlas coordinates. Following a craniotomy performed under sevoflurane anesthesia, we used topographic features of cortex to refine the location of the hand representation. Two 32-channel N-Form Modular Bionic (Santa Clara, CA, USA) arrays were implanted subdurally in the right post central gyrus, medial to the terminus of the intraparietal sulcus. The arrays consisted of a 2 × 2 arrangement of probes, with seven electrode sites per probe ranging from 2 to 3.5 mm in depth. Each probe also had a shallow electrode site 1 mm into the cortex used as an electrode reference (Figure 3 inset). The depth of these probes indicates likely area 1 placement, with potential area 3b at the deepest sites.

We used a set of novel, fascicle specific targeted longitudinal intrafascicular electrode (FAST-LIFE) arrays with integrated cuff electrodes for peripheral nerve stimulation, designed by Nerves Incorporated (Dallas, TX, USA). Each array was built with nine intrafascicular sites and six cuff sites composed of laser cut platinum suspended in a silicone mesh. Intrafascicular electrodes allow for precise recruitment and the cuff electrodes provide the opportunity to stimulate larger populations. Targeted microsurgical dissection of peripheral nerve fascicles ensures that the intraneural electrodes penetrate fascicles with desired sensory and/or motor functions. This design and strategy has seen success in human subjects, with respect to stability, recruitment, and sensation (Overstreet et al., 2019). In NHP-R’s left arm, arrays were implanted in the lateral cord contribution (LCC) and medial cord contribution (MCC) of the median nerve.

Connecting to the subcutaneous arrays requires the manufacturing of a custom percutaneous housing. To ensure stability, the housing was mounted onto an osseointegrated bone plate on the left humerus of NHP-R. Bone plates and housing components were manufactured *via* laser-sintered titanium with high resolution and acceptable biocompatibility (ProtoLabs, Maple Plain, MN, USA). This provided easy access and secure chronic housing for the connectors. To reduce the opportunities for damaging the device and the implantation site, NHP-R wore a fitted custom jacket from Lomir Biomedical (Notre-Dame-de-l’Île-Perrot, QC, Canada).

### 2.4. Neurophysiological interface

Recordings and stimulations were performed with a Grapevine Neural Interface Processor from Ripple Neuro (Salt Lake City, UT, USA) and custom software developed in MATLAB (Natick, MA, USA). Cortical electrodes were recorded at 30 kHz through Ripple Micro+Stim front ends, with no filters applied. Peripheral electrodes were stimulated *via* Ripple Nano2 front ends, which allow in-vivo impedance measurements and varied stimulation current resolution. Impedance values were stored at the beginning of stimulation sessions.

### 2.5. Stimulation

Two modes of mechanical stimulation were delivered to the fingertips of NHP-R’s left hand: vibrotactile and punctate. Force equivalent vibratory stimulation was delivered *via* a translating probe attached to a speaker mounted Bowden cable. Vibration frequencies were delivered from 10 Hz to 35 Hz on 5 Hz intervals. Frequency signals were generated in MATLAB and delivered through a translating probe attached to a vibration generator (3B Scientific). Signal amplitude was calibrated to ensure equivalent average force of the probe across frequencies. Punctate stimulation consisted of 0.1 to 1 mm depth, on intervals of 0.15 mm. Stimulation was delivered *via* a servo-mounted cable in a custom housing, with translation distance calibrated to the servo rotation. In both cases, the fingertip rested against the device actuation point and stimulation lasted for a 0.5 s window of each trial. Both modes also had “No Stimulation” and “No Contact” control conditions.

As this task is passive, there is an inherent risk that the area of cortex implanted will not respond to electrical stimulation even if a percept is present. Any of the NHP’s percepts could be potentially nociceptive or unpleasant. To avoid the risk of causing distress and to avoid NHP-R becoming agitated, electrical stimulation was limited to lower frequencies and lower amplitudes. After initial testing of variant responses and NHP-R sensitivity, stimulation was kept below 250 μA, consistently performed for 0.5 s at 40 Hz frequency, and consisted of 500 μs wide anodal leading charge balanced pulses.

Because the participation of NHP-R was passive, psychophysical detection thresholds were not obtained. Instead, cortical responses to varied current amplitude were evaluated with the Parameter Estimation by Sequential Trial (PEST) method (Taylor and Creelman, 1967). This method sequentially narrows in on the parameter of choice using the “positive” or “negative” detection results of a previous trial. In this task, “positive” responses occurred when multiple cortical channels demonstrated 100% RMS increase of the voltage response within the stimulation window compared to a baseline window. This was determined online after each trial. These current amplitude levels for each channel are described as Activity Thresholds (AT) and were used to choose appropriate values for stimulation schemes.

**FIGURE 1:**
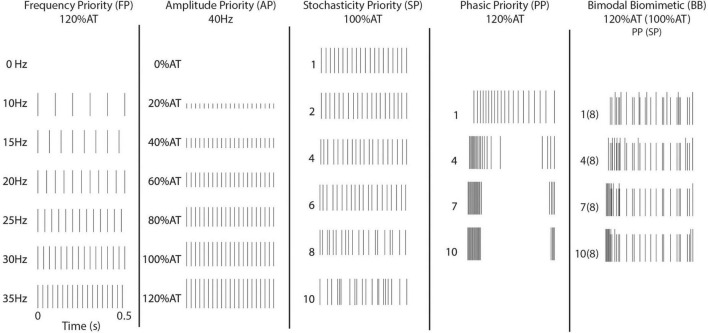
Stimulation patterns. Illustrated are the timing patterns associated with stimulation. The BB stimulation uses the 8th level of SP across the four levels of PP.

Five different timing schemes were considered, based on existing stimulation paradigms and peripheral/cortical activity patterns. Basic stimulation patterns were used to investigate the commonly induced percepts. First, Frequency Priority (FP) stimulation was delivered at 10–35 Hz on intervals of 5 Hz. Amplitude was chosen to be 120% of the respective channel’s determined activity threshold (120% AT). Second, Current Priority (CP) stimulation was delivered at 40 Hz from 0 to 120% AT on intervals of 20% AT (Figure 1).

In the remaining stimulation schemes, time variant pulses were introduced. In these, the 40 Hz frequency of stimulation was redefined as the average number of pulses per second. Four levels of Phasic Priority (PP) stimulation were performed at 120% AT. PP stimulation consists of increasingly clustered onset-release bursts at the start and end of the stimulation train, biased toward onset using a combination of exponential functions. Next, six levels of Stochasticity Priority (SP) stimulation were delivered at 100% AT. Stochasticity here is defined as increased randomization of interpulse intervals. Equations to calculate these stimulation schemes are available in the Supplementary Text and illustrated in Supplementary Figure 1 (Phasic Priority) and Supplementary Figure 2 (Stochasticity Priority). The final stimulation strategy results from the combination of four levels of PP and a single SP level. This combinatory scheme is referred to as Bimodal Biomimetic (BB) stimulation. It consists of the varied onset-release clustering of PP at 120% AT combined with a consistent level 8 pattern of SP at 100% AT. After combining patterns, we ensured no timings would cause the bimodal pulse to overlap.

For each stimulation session, it was important to keep train patterns consistent between all trials. While timing randomization is sometimes involved in the construction of the train, there is no inter-trial variation as generated timing patterns were maintained and reused. Stimulations patterns were defined at the onset of the experimental sessions and timing kept consistent between all deliveries of each trial. This allowed us to infer responses as results of timing principles and not inter-trial variation of the pulse timing.

### 2.6. Analysis

**FIGURE 2:**
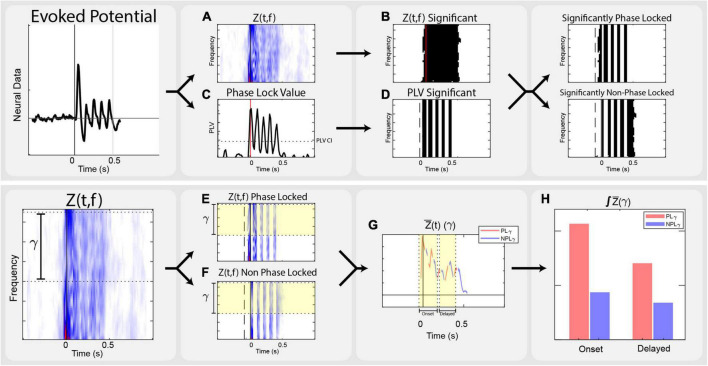
Analysis flow. Outline of the steps taken in analysis on a vibrotactile trial. **(A)** Transform evoked potentials into event related power, corrected by the standard deviation of the baseline power of each frequency, Z(t,f). **(B)** Use non-parametric analysis to determine significant time frequency points across stimulation conditions, Z_sig_(t,f). **(C)** Calculate Phase Lock Value, PLV(t,f), across trials of each condition. **(D)** Determine time points of significant phase locking by calculating the Phase Lock Value Confidence Interval, PLV_CI_. **(E)** Calculate the PLγ by isolating the average Z_γ_ (t) that is stimulation significant and PLV significant. **(F)** Calculate the NPLγ by isolating the average Z_γ_ (t) that is stimulation significant and not PLV significant. **(G)** Separate data into onset [–50 to 150 ms] and delayed [200 to 400 ms] epochs. **(H)** Integrate significant gamma states within onset and delayed time domains to compare to the perceptually appropriate standard.

Analysis focused on the local field potential recordings from the cortical arrays, relying on time-frequency transformations of continuous traces. Neural recordings were performed at 30 kHz in 1.5-second-long trials. Offline, the data were filtered through an 8th order 1,000 Hz low pass bidirectional filter, and down sampled to 3,000 Hz. The overall analysis process is illustrated in Figure 2.

Local field potential data were analyzed using the Chronux and FieldTrip toolboxes in MATLAB. Trials of each stimulation condition were linearly detrended and filtered in Chronux. Fourier transforms, phase locking calculations, and non-parametric statistics were performed in FieldTrip. To achieve adequate spectral estimates in both frequency and time resolution, a multitaper transform using 3 discrete prolate spheroidal sequence or Slepian window (DPSS) tapers with a fixed 50 ms timestep across the stimulus trial. Multitaper spectral estimation uses pair-wise orthogonal tapers to obtain independent estimates from the same sample, providing more reliable estimations of single trial spectral power without losing inter-trial variability. This yields a complex time-frequency spectral estimate for each point of the trial time course. We computed spectral power between 25–70 Hz, but focused analysis between 30–65 Hz. Event related Z-scores, Z(t,f) corrected to the baseline window from −0.5 s to −0.1 s were used to evaluate stimuli responses. This scales each frequency’s power to the standard deviation of its respective baseline (Eq. 1) Configuration parameters for multitaper spectral estimation in FieldTrip are included in Supplementary Text.

Significant time-frequency clusters for each stimulation location and modality were determined by non-parametric analysis using a point by point comparison across stimulation levels (Maris and Oostenveld, 2007). This produced a time-frequency F-statistic representation across respective stimulation levels. We compared those distributions against a nonparametric distribution constructed by 1,500 random permutations of the trials. Using α < 0.05, a critical value was determined for each time-frequency point, resulting in spectral power maps showing time-frequency significance that was corrected for the family wise error rates and addresses the multiple comparison problem: Z_*sig*_(t,f). This provides the ability to investigate time-frequency clusters that are significantly responsive to stimuli. Only significant points of Z(t,f) within the gamma band of 30–65 Hz were considered.


(1) $$\text{Z}\,\left(t_{\gamma \,S\,i\,g},f\right)=\left[P\,\left(t_{\gamma \,S\,i\,g},f\right)-P\,\left(t_{b\,a\,s\,e,f}\right)\right]/\sigma \,\left(t_{b\,a\,s\,e,f}\right)$$

In addition, the phase synchrony of each stimulation condition was measured by calculating the Phase Lock Value (PLV) across all relevant trials (Lachaux et al., 1999; Roach and Mathalon, 2008). PLV is an estimation of the consistency of phase (Eq. 2) and used to identify the significant components of the time-frequency representation that were phase locked to stimulation: significant phase locking is indicated by PLV measures that exceed a 95% confidence interval PLV_*CI*_, calculated by repeating the PLV calculation using trials of all conditions and randomly permuted 5,000 times across cortical electrodes. After averaging across gamma frequencies, the maximum value represents the PLV_*CI*_. In other words, the maximum PLV of sufficiently random trials represents the minimum PLV level for trials that are significantly phase locked.


(2) $$P\,L\,V\,\left(t,f\right)=\left|\frac{1}{N}\,\sum_{n=1}^{N}\frac{Z_{n}\,\left(t,f\right)}{\left|Z_{n}\,\left(t,f\right)\right|}\right|$$

In order to dissect the phase locked gamma and non-phase locked gamma, the time-domain significance of PLV was used to create a second time-frequency mask. Since the PLV confidence interval is calculated as the average across all gamma frequencies, this mask applies to time points across all frequencies. To alleviate comparison issues between these two masks, power across the frequency domain is averaged, followed by integration of power along the time domain. Averaging across the significant points of the power band equates activity in any frequency of the band. Averaging across significant time points would equate trials that had a single significant time point or significance across the entire window. Integrating across time ensures trials with more significance in time are given more weight. This assigns equivalence for magnitude of power anywhere in the gamma range and accounts for varied activity within time domain; significant gamma activity at a single time point is not equivalent to a span of time. Averaging in the time domain, significant Z(t,f) with PLV above the confidence interval are considered phase-locked (Eq. 3). Conversely, the non-phase locked gamma is the frequency-averaged and time-integrated gamma where the time-frequency ANOVA is significant and the time domain PLV is not significant (Eq. 4).


(3) P⁢L⁢γ=∫Z⁢(t,f),f=γ,

$$t\in \left\{\left[\left(\frac{1}{N_{f}}\sum_{f=\gamma }PLV\left(t,f\right)\right)>PLV_{C\,I}\right]\cap \left[Z_{s\,i\,g}\left(t,f\right)\right]\right\}$$

(4) N⁢P⁢L⁢γ=∫Z⁢(t,f),f=γ,

$$t\in \left\{\left[\left(\frac{1}{N_{f}}\sum_{f=\gamma }PLV\left(t,f\right)\right)\leq PLV_{C\,I}\right]\cap \left[Z_{s\,i\,g}\left(t,f\right)\right]\right\}$$

Proper magnitude estimation of separate phase locked and non-phase locked gamma in the time domain allows for the investigation of inter-trial variance. Across multiple sensory systems, the immediate onset of gamma is phase locked to stimuli. For this reason, we separated analysis into onset and delayed time domains. Due to the windowed nature of time-frequency analysis, onset time includes data bounding the stimulus by −50 ms to 150 ms. The delayed time domain, from 200 ms to 400 ms, is designed to encompass the existing representation of cortical activation seen in perception-related gamma band sensory literature.

Using the evoked potential responses and the quantitatively determined gamma phase-locking characteristics in the onset and delay domains, responses can be classified if aligned with “Appropriate Response in the gamma band” (ARγ) traits. These characteristics are defined as any qualities observed in the cortical response to mechanical punctate stimulation that differ from vibrotactile stimulation, creating a rubric on which to evaluate peripheral nerve stimulation. The concurrence of such ARγ traits in peripheral nerve stimulation responses suggests support of a more appropriate stimuli that avoids vibratory percepts.

## 3. Results

### 3.1. Significant cortical channels

**FIGURE 3:**
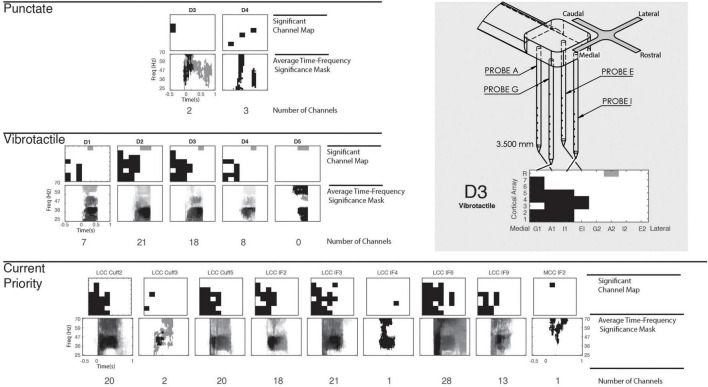
Cortical significance to mechanical and peripheral nerve stimulation. Cortical array structure and representation illustrated in the top right. The N-Form cortical arrays consist of a 2 × 2 arrangement of probes, with seven recordings sites and a single shallow reference. Two arrays are implanted into somatosensory cortex, with direction defined in the figure. Included is an example response of the array to illustrate the medial-lateral and depth arrangement of the figure. On the left, significant cortical channels are shown for punctate, vibrotactile, and CP stimulation, but significant REF channels of the lateral array are indicated in gray. Below the cortical electrode maps, time-frequency significance masks are constructed by averaging the Boolean time-frequency mask (Figure 2B) for each significant channel. This shows common points of response across all significant cortical channels. All stimulation types evoke significant responses predominantly on the medial array.

To appropriately compare cortical responses between mechanical stimulation and peripheral stimulation on different digits and electrodes, the channels with significant cortical activation were first determined. These channels were identified *via* a nonparametric point-by-point ANOVA across response spectrograms (Figure 2A), determining any significant time-frequency points within the gamma frequency band and within the trial window (Figure 2B). Figure 3 provides representations of which cortical channels significantly respond to punctate, vibrotactile, and CP stimulation for each digit/stimulation electrode. Cortical channels with a significant response to CP stimulation were used for all peripheral nerve stimulation representations, as they indicate neuronal recruitment after adequate stimulus intensity. Average time-frequency significance mask across the significant electrodes are shown in Figure 3.

Mechanical stimulation produced variable responses across digits between stimulation modes. Punctate stimulation was very localized: mechanical stimuli to D3 and D4 produced specific responses in few cortical channels. The second array, implanted more medially, consistently had limited, if not absent, responses. Vibrotactile stimulation had more robust and deeper activation across the medial array, with no lateral significance except shallow reference electrodes. Stimulation of D2 recruited the most channels, with D3 being similarly responsive. Diminished responses were present for D1 and D4, but not D5. A control stimulation, where the mechanical stimulator did not contact any digit, produced no significant responses in either mechanical mode.

Significance masks resulting from the CP stimulation provide the time-frequency response of interest for all peripheral nerve stimulation patterns (Figure 3). In all cases, we found significant cortical response to peripheral nerve stimulation. The number of cortical channels which responded significantly to individual peripheral nerve channels ranged from 1 to 28 and averaged 12 channels, with broad time-frequency significance. In most cases, cortical channels with significant responses to the peripheral nerve stimulation encompassed the cortical response to mechanical stimulation, especially vibrotactile. Only one MCC stimulation channel produced a significant response, and only in one cortical channel.

### 3.2. Evoked potentials

**FIGURE 4:**
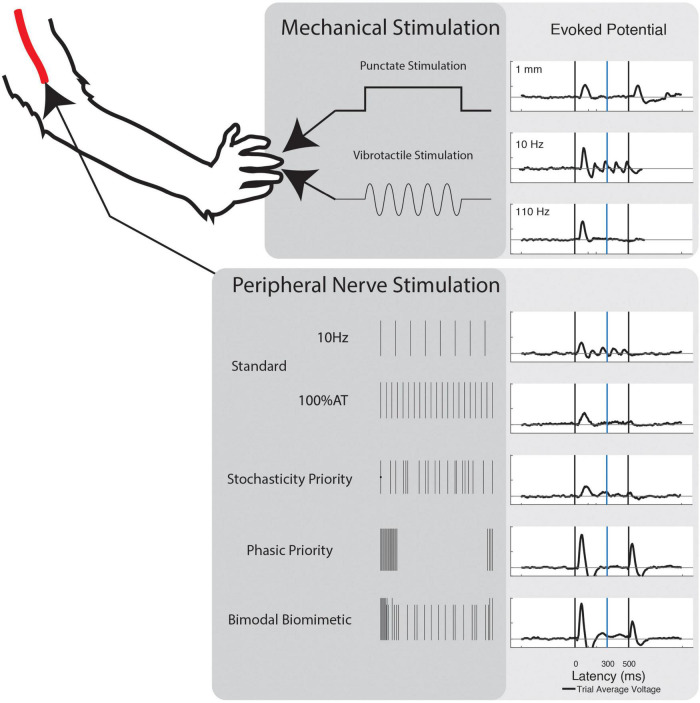
Evoked cortical response of each stimulation mode. Evoked potentials for all modes of stimulation. Punctate stimulation exhibits evoked peaks after stimulation start and stop. This is mimicked by peripheral nerve stimulation only when the stimulation includes the phasic patterns. During both vibrotactile and constant frequency stimulation, cyclic entrainment is obvious. This diminishes at higher frequencies, but the evoked “off” response is absent.

Using the selected significant channels, we averaged the evoked potentials for each stimulation mode and respective increments. From this, typical response traits become obvious for digits and stimulation electrodes (Figure 4). Punctate stimulation produced strong positive fluctuation near 125 ms and a negative fluctuation near 175 ms. There is a slight positive inflection near 250 ms at high magnitudes of punctate stimulation but dwarfed by the initial response. Upon retraction of the punctate stimulus, we observed a release response: the evoked response repeated as if in response to a second distinct stimulation. The presence of a 250 ms positive fluctuation seen in the onset evoked response is not present in the release evoked response. Vibrotactile stimulation at low frequency consistently demonstrates the 125 ms and 175 ms fluctuations as well. The initial response is followed by repeated evoked peaks concurrent with stimulation frequency. Unlike punctate stimulation, there was no release response to vibrotactile stimulation. This is an important distinction between the two stimulus.

The evoked potentials across all peripheral nerve stimulation electrodes follow similar patterns, with varied magnitudes of responses. All stimulations, except CP at low levels, demonstrated a strong positive potential at 75 ms consistently followed by a negative inflection. While FP and CP have increased activity at 250 ms, there is no obvious peak and there is visually identifiable entrainment of the stimulus. A 250 ms potential, within the P300 range, is clear for time variant patterns SP, PP, and BB. The only demonstration of release evoked potentials occurs in PP and BB around 50 ms after stimulation. BB also demonstrates a higher P250 response than PP or SP. In terms of evoked potentials, constant frequency stimulations seem comparable to the vibrotactile stimuli, while the stimulation pattern most comparable to punctate stimulation is the composite bimodal biomimetic scheme.

The observation of EEG P300 responses can be teased apart into the P3a and P3b components, which dive further into discrimination and novelty, usually enhanced by novelty of the first stimuli in a matching sequence. As our experiment presents stimuli in randomized order with variable inter-trial intervals and no discrimination is tasked to the NHP, we make no claims into this segregation intracortical response timing but recognize the importance of investigating precise evoked response timing when tied to human perception. It would be possible the P3a to novel stimuli paradigms may be pronounced and diminish over training experience, regulating as perception interpretation stabilizes.

### 3.3. Gamma phase locking

**FIGURE 5:**
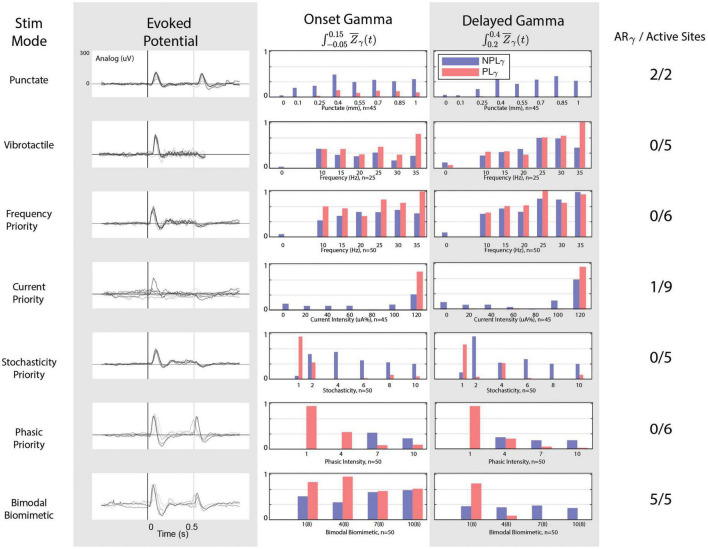
Cortical responses to stimulation modes. From left to right: stimulation mode, evoked potential responses, onset gamma phase locking, delayed gamma phase locking, and the number of stimulation electrodes/sites that possessed the “appropriate” response in terms of gamma and evoked responses. The onset-release evoked potentials peaks are noticeable only under punctate, PP, and BB stimulations. Only one CP channel but all BB stimulation channels achieve an absence of delayed phase locked gamma. All channels of SP and PP fail to achieve the release evoked response. BB stimulation achieves the appropriate gamma patterns and evoked activity for 5/5 active stimulation channels.

Using the analysis outlined in Figure 2, we determined the effects of stimulation mode on gamma response power, gamma response timing, and phase lock value. Using these metrics, the punctate stimulation cortical responses are most comparable to the bimodal biomimetic stimulation’s responses (Figure 5). Traits of the response to mechanical stimulation illustrate what we are calling the Appropriate and Inappropriate Gamma Responses. Punctate stimulation demonstrates a correlation between increased indentation magnitude and the delayed gamma power. Phase Locking Value calculations cross the 95% confidence interval near the onset and release of all punctate stimulation, with proportional magnitude. By contrast, vibrotactile gamma exhibits phase locking significance at onset, at 200 ms, and more frequently, sustained throughout stimulation. This is especially noticeable at 35 Hz stimulation, with the entire stimulation window demonstrating significant phase locking.

Using the time-frequency significance mask (Figure 2B) and the Phase Locking Value mask (Figure 2D), the event related power can be dissected into phase locked (Figure 2E) and non-phase locked gamma (Figure 2F): Z_*sig*_(t,f) and PLV(t) > CI or Z_*sig*_(t,f) and PLV(t,f) < = CI. Averaging across the gamma band within these masks results in separate non-continuous time series (Figure 2G). The classification as a response as either appropriate or inappropriate relies on the midpoint estimation integral of these time series within defined onset and delayed time. The key differentiator between these two is the presence of delayed gamma phase locking (Figure 2H).

For each stimulation mode, gamma activity trends between the onset and delayed window are shown in Figure 5. In the case of mechanical stimuli, significant responses to each digit are consistent with both PLγ and NPLγ for both punctate and vibrotactile stimuli. In the delayed time window, however, responses to mechanical stimuli exhibit an almost complete absence of PLγ during punctate stimulation. Therefore, punctate stimulation remains consistent with the ARγ trends described previously and remains the target for peripheral nerve stimulation responses. For all vibrotactile stimulations, NPLγ is present in the delayed window alongside PLγ. The phase locking effect becomes more pronounced with increase in frequency. In no cases is the cortical response to vibrotactile stimulation comparable to the punctate response.

For peripheral nerve stimulation, illustrated responses are from the LCC Intrafascicular-3 channel, but all stimulation channels with significant cortical responses are considered as active sites. FP stimulation demonstrates gamma patterns remarkably similar to vibrotactile responses: graded activation with frequency but abundant PLγ in the delay window. The CP stimulation demonstrates low gamma activity low in both temporal windows, even when onset PLγ appears appropriate, delay PLγ is also present. No stimulation channels achieve the desired ARγ target with FP or and only one with CP stimulation (LCC Cuff-3) as delayed phase locked gamma is abundant. SP fails to achieve the ARγ target on any channel as higher levels of SP drastically reduce phase locking in the onset window as well as the delayed window, but consistent NPLγ activation is achieved and an inflection around 250 ms is observed in the evoked response. When considering the desired aspects of the evoked response to FP, CP, or SP: all channels fail as none produce the desired release peak. While PP begins to demonstrate the appropriate relationship between NPLγ and PLγ, it fails to produce any evoked response around 250 ms. The cortical response to BB stimulation possesses the most similarity to the punctate stimulation’s response as five channels achieve the ARγ target and all five of those channels also possess the desired evoked potential traits as well. As PP grouping is increased for the onset-release responses, the addition of SP tonic responses provides the necessary sustained responses and desire phase locking dynamics.

## 4. Discussion

### 4.1. Natural response gamma definition

We report here that a stimulus pattern that combines onset-release bursts with sustained stochastic stimulation produces a cortical response that is similar to that evoked by a punctate stimulus. A crucial characteristic of this response is enhanced gamma activation throughout the stimulus, and phase locking only at the onset and release phases of the response. Other patterns of stimuli provoke cortical responses which differ in crucial ways. Namely, both vibrotactile stimulation and electrical stimulation with a fixed frequency produces phase-locking in the gamma band for the duration of the stimulus. This may be why the sensation evoked by electrical stimulation is so frequently described as buzzing, vibration, or tingling in humans (Clippinger et al., 1974; Walker et al., 1977; Gasson et al., 2005). In literature, painful stimuli can produce a similar profile, with phase-locking through the duration of the stimulus.

In this discussion, it is primarily important to distinguish two assumptions. One key feature of the appropriate response gamma is the presence or absence of delayed phase locking gamma. Literature suggests a defined and supporting timing structure of typical phase locked and non-phase locked gamma in multiple modes of sensory perception. The visual, auditory, tactile, and pain systems exhibit this pattern in remarkably similar manners. An appropriate gamma response manifests as onset phase locked gamma and increased delayed non-phase locked gamma near 250–300 ms after stimuli. Attention to and perception of a stimulus enhances any delayed non-phase locked gamma. For this reason, a strong inattention or weak perception would not produce a robust increase in the delayed response. This would likely not indicate an atypical sensation as this inattention-associated decrease is not accompanied with an increase in phase-locked response; therefore, only violation in typicality would be the presence of significant delayed phase locking. In terms of delayed gamma, literature only provides evidence that pain responses elicited from peripheral nerve stimulation and from electrotactile stimulation exhibit gamma phase-locking in the tonic phase (Babiloni et al., 2001; Rossiter et al., 2013).

Attention to stimuli is not important to demonstrate these characteristics and classify responses. All the stimuli presented in this study were entirely passive, requiring no responses from the animal, and hence did not incorporate control of attention. In this paradigm, we have shown the deviation between the typical gamma pattern of normal somatosensory activation and the observed violation from atypical non-painful mechanical stimuli. Then, the resulting deviation is applied to the results of varied patterns of median nerve stimulation. The goal is to identify stimulation parameters that modulate the violation of ARγ. All stimulations in this experiment are passive and require no perceptual response from NHP-R, as the goal is the cortical difference between stimuli and not the perceptual difference. The passivity suggests any augmentation of NPLγ responses within the time window of perceptual recognition are potentially a result of the primate attending a perceived stimulus. This is primarily seen in higher levels of current intensity, frequency, or time variant stimulations with biomimetic components. However, this puts the term “appropriate response gamma” into question. The non-attended task, presence of early evoked potentials and gamma, and the general lack of P300 evoked gamma indicate that the cortical representations observed are not likely representing attended stimuli or perceptual decisions. However, the comparison between responses is valid in terms of input to the somatosensory system. The definition of ARγ still applies, as the onset phase locking is only appropriately present in onset of perceived/attended and unperceived stimuli. The lack of delayed phase locking is also a key component of this definition. Therefore, this study is a strong investigation into the stimulation parameters, as the violation of appropriate input provides a predictive estimate of perception. The manipulation of this early cortical processing can contribute to the construction of desired percepts.

### 4.2. Peripheral electrode considerations

Cortical recruitment from peripheral stimulation is wider than expected with larger sets of significantly active channels present than in mechanical actuation. The intrafascicular stimulation was expected to provide smaller recruitments of neuronal populations. This could be a result of stimulation parameters chosen. The 500 μs pulse width is large, but the time required to sweep through all stimulation parameter sets was not available with this experiment. Once a set of parameters demonstrated little agitation on NHP-R, the stimulation timing experiments were performed.

Electrodes were implanted in the predominantly LCC and MCC of the median nerve. In the appendices, the impedances over this experimental window show a sharp increase at a month post implant for many channels across both arrays. This coincides with sharp change or loss in channels with determined activity thresholds. Upon explanation of these arrays, it was discovered that delamination occurred for all of the MCC implant and most of the LCC implant. This reduced the number of electrodes with complete data sets for analysis.

### 4.3. Successful modulation of gamma phase locking

Overall, the punctate stimulation demonstrates the predicted pattern of gamma phase locking activity. As the indentation magnitude increases, so does the delayed non-phase locked response and an evoked response peak at ∼300 ms eventually manifests. Other traits observed supplement the appropriate response definition by incorporating: (1) the presence of early onset-release evoked potentials, (2) the presence of significant onset-release phase locking, and (3) the sustained evoked gamma with no phase locking between the prominent onset and release patterns. Vibrotactile stimulation violated the ARγ definition in almost all cases by demonstrating sustained or delayed phase locking and no release response in terms of evoked potential, evoked gamma, or phase locking. Due to this, the violations of the defined rules do not necessarily indicate a painful response, but an atypical sensation of repeated bottom up activity. In summary, static tactile activation and dynamic tactile activation provide divergent cortical representations that provide necessary metrics for peripheral nerve stimulation comparisons.

Peripheral nerve stimulation behaved largely as expected, with standard stimulation patterns violating the ARγ responses. Consistent timing of pulses within the FP and CP, at adequate intensity, show persistent phase locking throughout the stimulation window. This is similar to the vibrotactile stimulation ARγ violations and provides evidence that the two modes activate the sensory system in similar methods. Hence, this is comparable to one of the most common elicited percepts in somatosensory stimulation: vibration and pulsing. If the cortical input representation can be modulated away from this similarity and maintain the effects of punctate stimulation, the perception of the stimuli may move away from these impractical sensations.

**FIGURE 6:**
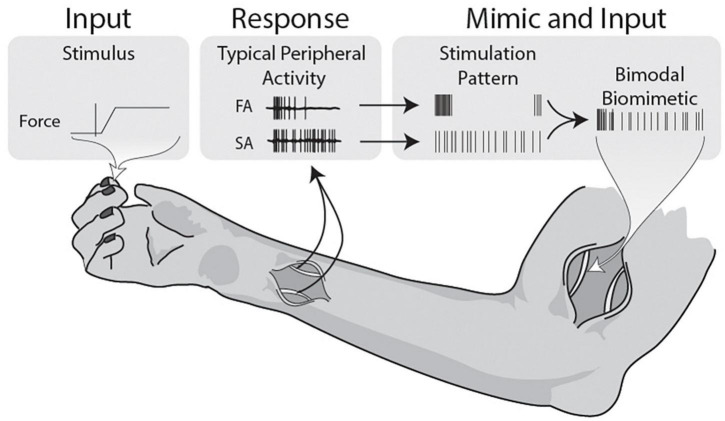
Illustrative summary of bimodal biomimetic construction. Force applied during active grip tasks and object manipulations activates both the slow and fast adapting mechanoreceptors in the skin. The patterns observed in the peripheral nerve demonstrate onset-release firing bursts and sustained firing, both with some level of stochasticity. Firing patterns are never composed of determinate evenly spaced pulses, so stimulation should aim to mimic the physiological data. This study uses stimulation patterns that individually mimic the FA and SA traits, and combines them into a composite stimulation. Using this basic model of Bimodal Biomimetic stimulation, cortical responses more similar to punctate stimulation can be achieved.

The different stimulation patterns with time variant properties modulate in manners consistent with this goal. Increasing interpulse variability with SP significantly decreases the delayed phase locking but loses the large onset and release evoked and PLV responses associated with punctate stimulation. However, the onset-release stimulation patterns do provide onset and release responses but lack the presence of delayed gamma or any hint of a 300 ms evoked inflection. By taking tonic and phasic patterns of physiological activity, mimicking them in stimulation, and implementing a composite stimulation paradigm of the two, a satisfying result is obtained (Figure 6). Capitalizing on the reduction in phase locking and sustained properties of the SP stimulation and the phase locking onset-release dynamics from the PP stimulation, cortical representations can be modulated into the conditions that satisfy appropriate response gamma. However, the beneficial combination relies on the balance between this onset-release stimulation and the sustained stochasticity, which needs to be optimized.

Exploration of this stimulation mode’s efficacy can be broken into each contributing component and their interaction. What properties of the onset-release bursts modulate the phase locked response most effectively? At present, the onset-release bursts differ in magnitude than the stochastic stimulation and are precisely timed with low interpulse variability. Continuation investigations should involve the effect of this magnitude ratio to determine what properties are necessary to achieve the onset-release phase locking in the presence of stochasticity. In the responses shown, the PP stimulation produced very large peaks of phase locking, while punctate stimulation produced smaller, albeit significant, peaks. It is likely that low magnitude PP can contribute the desired effect on a modulated scale. This would be analogous to the human touch, and how the rate of tactile sensation is not differentially perceived, but an important component of proper reactions.

Excessive stochastic noise can mask a signal, but optimized noise can boost the same signal. At optimal levels, the stochastic signal is randomly pushing the primary signal past the criterion of detection, providing the basic mechanism of stochastic facilitation. Results indicate that phase locking is reduced by neural noise, but non-phase locked data is also reduced. The absence of almost all PLγ at higher SP is consistent with high noise in a sensory system masking the primary signal. With optimization, the stochastic component needs to diminish phase locking but not incur masking of primary signals. Inclusion of optimized stochastic variance and the balanced current intensity ratio could work as an “on-demand” stochastic facilitation model. A primary signal consisting of the minimized onset-release phase locking represents rate of contact while the stochastic stimulation provides sustained gamma presence and increases sensitivity to tactile properties. Conceptually, this model would be a great benefit to active tasks in prosthetic users in just providing increased sensitivity only while contacting an object. If the actual percepts of the sensation manifest in more typical and practical tactile sensations, the result is profound.

### 4.4. Limitations

There are two primary limitations to address in this study. First, there is a gap between the perceptual potential and the observed cortical response differences between standard and biomimetic stimulation paradigms. There is ample pre-existing support and literature that link such cortical differences with distinct perceptual outcomes. Admittedly there is a gap between our data and perceptual outcomes. Nonetheless, we would argue that the striking similarities we observed in the cortical responses to our stimuli (FP vs. biomimetic) and physical stimulation (vibrotactile vs. punctate) provide strong motivation to test these stimulation schemes in humans.

Second, the present study was performed with a single *Macaca mulatta*, NHP-R, which presents a marked reservation on the interpretation of the data. However, results from this single NHP provide compelling and consistent results that are observed across multiple peripheral electrodes and across somatosensory intracortical channels. The high-level questions that arise about perception stability, manipulation, and dynamics would be better suited for human models of peripheral or electrotactile stimulation rather than continued NHP experiments.

## 5. Conclusion

The cortical response to punctate stimulation follows the same patterns of other sensory systems with regards to the presence and timing of phase locked and non-phase locked gamma. These patterns are violated by vibrotactile stimulation, confirming that pain is not the only form of stimulus that induces a cortical response with phase-locked gamma. Standard constant frequency stimulation patterns induce phase locked gamma, which could explain the common pulsing, vibration, and pain percepts often elicited in sensory feedback *via* peripheral nerve stimulation. The composition of a stimulation pattern based on neurophysiological tactile information is performed by combining tonic and phasic dynamics with stochastic elements. Using this pattern in median nerve stimulation achieves modulation from atypical responses into appropriate cortical representations. These lack delayed PLγ but maintain NPLγ and biomimetic onset-release evoked potentials. The variables of the bimodal stimulation model are not well investigated herein but provide a strong groundwork for future stimulation schemes in terms of practical sensation or practical benefit to tactile sensitivity.

## Data availability statement

The raw data supporting the conclusions of this article will be made available by the authors, without undue reservation.

## Ethics statement

The animal study was reviewed and approved by ASU IACUC.

## Author contributions

All authors supported the experimental design, analysis, and manuscript writing.
