# Supplementary material for: Dynamic peripheral nerve stimulation can produce cortical activation similar to punctate mechanical stimuli
Source: Front Hum Neurosci. 2023 Mar 24;17:1083307. doi: 10.3389/fnhum.2023.1083307 (PMC10079952; doi:10.3389/fnhum.2023.1083307)
Supplement: Supplementary file 1 [file Data_Sheet_1.docx]

**Supplemental Materials**

Onset Release Stimulation Equation

$$Given:$$

$$TD:Train Duration$$

$$t:time from 0 to TD$$

$$M:Magnitude of Modulation \left( 0 to 10 \right)$$

$$f:Stimulation Pulses per Second$$

$$Generate Relative Probability of Pulse$$

$a\left( t \right)=\frac{t}{TD}e^{-5*M*\frac{t}{TD}}+\left( TD-t \right)*e^{-15*M*\left( TD-t \right)}$, $\hat{a}\left( t \right)= \frac{a(t)}{\left\| a(t) \right\|}$

$$Generate Normalized Cumulative Summation of Probability$$

$A\left( t \right)=\sum_{t=0}^{t=TD} \hat{a}(t)$, $\hat{A}\left( t \right)=\frac{A(t)}{\left\| A(t) \right\|}$

$$Stimulation Pulse Times at 't' where$$

$$\hat{A}(t)=\left[ \frac{1}{f*TD} :\frac{1}{f*TD}:1 \right]$$

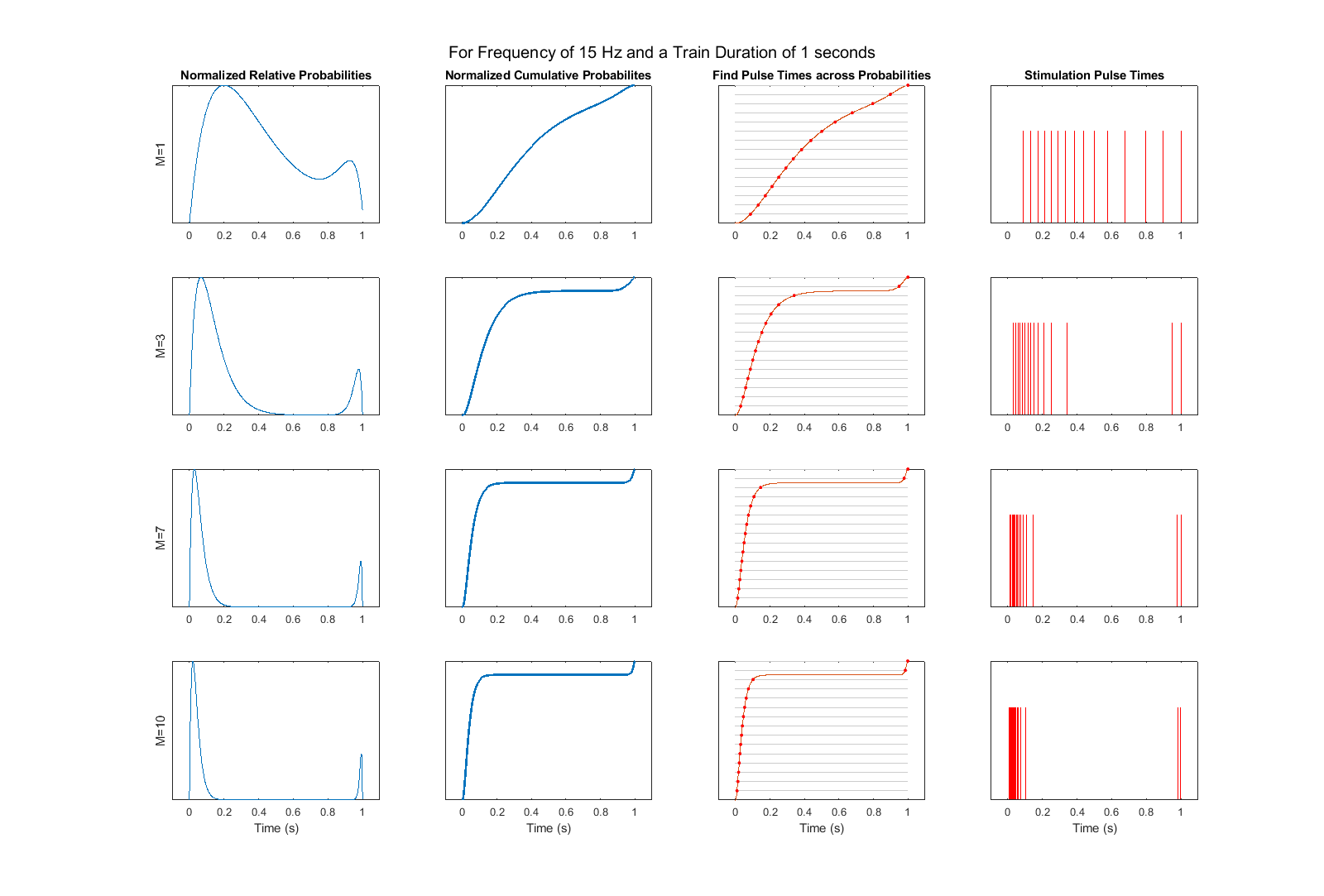


Supplemental Figure 1. Illustration of Onset-Release Stimulation Calculations. On the left is the normalized relative probabilities that a stimulation pulse should occur at that point in the stimulation train for each magnitude of modulation. The next is a normalized cumulative summation of probabilities so that probabilistically evenly spaced pulses can be determined, illustrated in the third column. The time points of those probabilities are illustrated in the final column.

Stochastic Stimulation Equation

$$Given$$

$$TD:Train Duration$$

$$t:time from 0 to TD$$

$$M:Magnitude of Modulation \left( 0 to 10 \right)$$

$$f:Stimulation Pulses per Second$$

$$PW:Pulse Width$$

$$Define Range of Stochastic IPI and Generate a set for all desired pulses$$

${}_{1}^{\left\lfloor f*TD \right\rfloor}{IPI(n)}=R \left\{ \begin{aligned} \frac{1+\frac{M}{10}}{f} \\ \frac{1-\frac{M}{10}}{f} \end{aligned} \right.$ , $where \left[ \frac{1-\frac{M}{10}}{f}>\left( 2*PW \right) \right]$

$$so that \left( TD-\frac{1}{f} \right)\leq\left[ \sum_{n=1}^{\left\lfloor f*TD \right\rfloor} IPI(n) \right]\leq\left( TD+\frac{1}{f} \right)$$

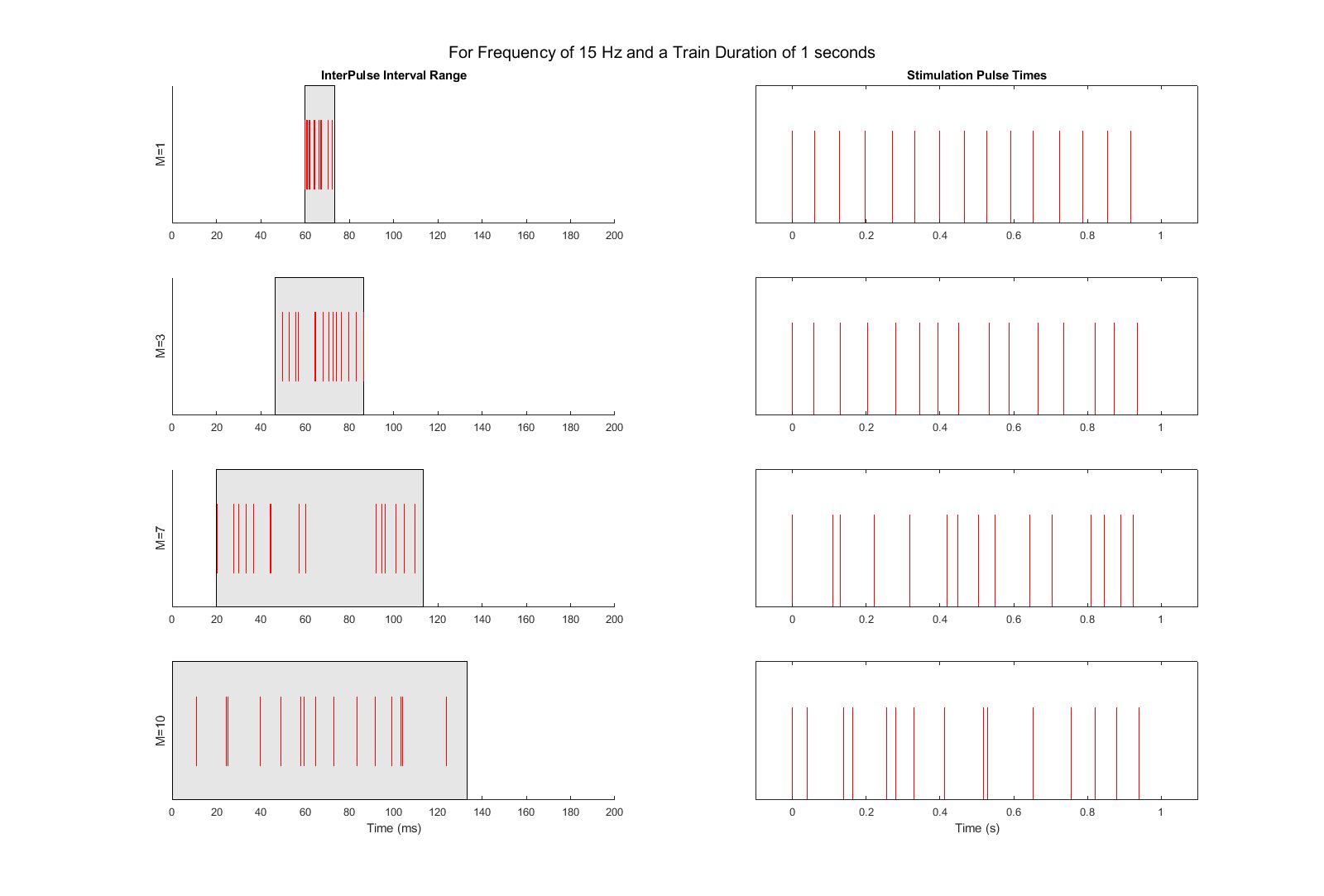


Supplemental Figure 2. Illustration of Stochastic Stimulation Calculations. On the left is the window of interpulse intervals that can be randomly sampled for each magnitude of modulation, and example pulses sampled from within that window. Once the summation of these randomly sampled intervals exists within the criteria, the time points are displayed in the right column.
