# Supplementary material for: Dynamic peripheral nerve stimulation can produce cortical activation similar to punctate mechanical stimuli
Source: Front Hum Neurosci. 2023 Mar 24;17:1083307. doi: 10.3389/fnhum.2023.1083307 (PMC10079952; doi:10.3389/fnhum.2023.1083307)
Supplement: Supplementary file 2 [file Data_Sheet_2.docx]

Supplementary Text

# FieldTrip Configuration Parameters for ‘ft_freqanalysis()’ Function

cfg.foi= [25:70];
cfg.toi = -.5:.05:1;
cfg.t_ftimwin = 6.5./cfg.foi;
cfg.tapsmofrq = cfg.foi*0.2;
cfg.taper = 'dpss';
cfg.baseline = [-(0.5) -.1];
cfg.method = 'mtmconvol';
cfg.pad = 'nextpow2';
cfg.output = 'fourier';
cfg.keeptrials = 'yes';
cfg.parameter='fourierspctrm';
cfg.polyremoval = 1;
cfg.baselinetype = 'standarddevscale';
